# Right ventricular volume and function by three‐dimensional echocardiography: results of the echocardiographic measurements in normal Chinese adults (EMINCA) II

**DOI:** 10.1002/mco2.550

**Published:** 2024-04-21

**Authors:** Yu Zhang, Ying‐Bin Wang, Gui‐Hua Yao, Hong Tang, Li‐Xin Chen, Li‐Xue Yin, Tian‐Gang Zhu, Jian‐Jun Yuan, Wei Han, Jun Yang, Xian‐Hong Shu, Ya Yang, Yu‐Lin Wei, Yan‐Li Guo, Wei‐Dong Ren, Dong‐Mei Gao, Gui‐Lin Lu, Ji Wu, Hong‐Ning Yin, Yu‐Ming Mu, Jia‐Wei Tian, Li‐Jun Yuan, Xiao‐Jing Ma, Hong‐Yan Dai, Yun‐Chuan Ding, Ming‐Yan Ding, Qing Zhou, Hao Wang, Di Xu, Mei Zhang, Yun Zhang

**Affiliations:** ^1^ State Key Laboratory for Innovation and Transformation of Luobing Theory, Key Laboratory of Cardiovascular Remodeling and Function Research, Chinese Ministry of Education, Chinese National Health Commission and Chinese Academy of Medical Sciences, Department of Cardiology Qilu Hospital of Shandong University Jinan China; ^2^ Department of Cardiology Qilu Hospital of Shandong University (Qingdao) Qingdao China; ^3^ Department of Ultrasonography West China Hospital, Sichuan University Chengdu China; ^4^ Department of Ultrasonography Shenzhen People's Hospital/The Second Clinical Medical College of Jinan University Shenzhen China; ^5^ Department of Ultrasonography Electronic Science and Technology University of China, The Affiliated Sichuan Provincial People's Hospital Chengdu China; ^6^ Department of Cardiology Peking University People's Hospital Beijing China; ^7^ Department of Ultrasonography Henan Provincial People's Hospital Zhengzhou China; ^8^ Department of Cardiology The First Affiliated Hospital of Harbin Medical University Harbin China; ^9^ Department of Echocardiography The First Affiliated Hospital of China Medical University Shenyang China; ^10^ Department of Echocardiography Zhongshan Hospital, Fudan University Shanghai China; ^11^ Department of Echocardiography Beijing Anzhen Hospital, Capital Medical University Beijing China; ^12^ Department of Cardiology Sun Yat‐Sen Memorial Hospital, Sun Yat‐Sen University Guangzhou China; ^13^ Department of Ultrasonography The Southwest Hospital of AMU Chongqing China; ^14^ Department of Ultrasonography Shengjing Hospital of China Medical University Shenyang China; ^15^ Department of Ultrasonography China‐Japan Union Hospital of Jilin University Changchun China; ^16^ Department of Ultrasonography First Affiliated Hospital, School of Medicine, Shihezi University Shihezi China; ^17^ Department of Ultrasonography The First Affiliated Hospital of Guangxi Medical University Nanning China; ^18^ Department of Echocardiography The Second Hospital of Hebei Medical University Shijiazhuang China; ^19^ Department of Ultrasonography The First Affiliated Hospital of Xinjiang Medical University Urumqi China; ^20^ Department of Ultrasonography The Second Affiliated Hospital of Harbin Medical University Harbin China; ^21^ Department of Ultrasonography Tangdu Hospital of Air Force Medical University of PLA Xi'an China; ^22^ Department of Ultrasonography Wuhan Asia Heart Hospital Wuhan China; ^23^ Department of Cardiology Qingdao Municipal Hospital Qingdao China; ^24^ Department of Ultrasonography Yan'an Hospital Affiliated to Kunming Medical University Kunming China; ^25^ Department of Ultrasonography The People's Hospital of Liaoning Province Shenyang China; ^26^ Department of Ultrasonography Renmin Hospital of Wuhan University/ Hubei General Hospital Wuhan China; ^27^ Department of Ultrasonography Fuwai Hospital/Chinese Academy of Medical Sciences Beijing China; ^28^ Department of Ultrasonography Jiangsu Province Hospital Nanjing China

## Abstract

Three‐dimensional (3D) echocardiography is an emerging technique for assessing right ventricular (RV) volume and function, but 3D‐RV normal values from a large Chinese population are still lacking. The aim of the present study was to establish normal values of 3D‐RV volume and function in healthy Chinese volunteers. A total of 1117 Han Chinese volunteers from 28 laboratories in 20 provinces of China were enrolled, and 3D‐RV images of 747 volunteers with optimal image quality were ultimately analyzed by a core laboratory. Both vendor‐dependent and vendor‐independent software platforms were used to analyze the 3D‐RV images. We found that men had larger RV volumes than women did in the whole population, even after indexing to body surface area, and older individuals had smaller RV volumes. The normal RV volume was significantly smaller than that recommended by the American Society of Echocardiography/European Association of Cardiovascular Imaging guidelines in both sexes. There were significant differences in 3D‐RV measurements between the two vendor ultrasound systems and the different software platforms. The echocardiographic measurements in normal Chinese adults II study revealed normal 3D‐RV volume and function in a large Chinese population, and there were significant differences between the sexes, ages, races, and vendor groups. Thus, normal 3D‐RV values should be stratified by sex, age, race, and vendor.

## Graphical abstract

A set of normal values of three‐dimensional right ventricular volume and function was first established in a large Chinese population, which was significantly smaller than those recommended by the international guidelines.

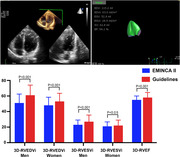

## 1 INTRODUCTION

Right ventricular (RV) function is the single most important prognostic determinant of survival in many cardiac and pulmonary diseases, such as heart failure, pulmonary hypertension, pulmonary embolism, and congenital heart disease.^1^, ^2^, ^3^ An ideal technique for measuring RV function should be noninvasive, accurate, reproducible, and convenient.^3^ Currently, cardiac magnetic resonance (CMR) imaging is the gold standard for measuring RV function but has several important limitations, including detrimental magnetic effects, time‐consuming image acquisition and analysis, high cost, and instrument unavailability in many hospitals.

Two‐dimensional (2D) echocardiography provides a noninvasive technique for imaging the right heart, but quantification of RV volume and function requires geometric assumptions, which are hindered by the crescentic and thin‐walled shape of the normal right ventricle, which cannot be imaged with a single 2D plane.^3^, ^4^, ^5^, ^6^ Three‐dimensional (3D) echocardiography is an emerging technique for assessing RV volume and function, but previous studies in this field have provided only small sample data or meta‐analysis results^7^ for white populations.^8^, ^9^, ^10^ Recently, a substudy on 3D‐RV volume and function was published in the World Alliance Societies of Echocardiography (WASE) Normal Values Study but included only 373 Asian subjects in whom the Chinese proportion was not reported.^11^ In addition, 3D echocardiography was deemed less reproducible than CMR for RV assessment according to a recent official statement of the American Thoracic Society.^3^ Thus, normal values of RV volume and function by 3D echocardiography in a large non‐white population are still lacking, and the reproducibility of these measurements is not satisfactory.

In 2015, the American Society of Echocardiography (ASE) and the European Association of Cardiovascular Imaging (EACVI) issued guidelines to standardize RV assessment with 3D echocardiography and recommended normal values of RV volume and function based on small sample studies in white populations.^12^ However, two fundamental problems remain unsolved. First, what are the definitions of “normal” and “abnormal” RV structure and function? Second, how do key demographic characteristics, such as age, sex, and race, affect the definitions of “normal” and “abnormal”?^3^ The echocardiographic measurements in normal Chinese adults (EMINCA) study was a prospective, nationwide, and multicenter study that reported normal values of 2D and Doppler echocardiography in healthy Chinese adults in 2015 and 2016.^13^, ^14^ The EMINCA II study was designed, organized, and conducted from September 2016 to February 2020, with the purpose of establishing normal reference values of LV and RV and atrial size and function by 3D echocardiography in a large cohort of healthy Han volunteers, and examining the physiological and instrumental factors that may affect these normal values.

In this study, we focused on the reference values of RV volume and function by 3D echocardiography, and provided normal values of 3D‐RV volume and function in a large Chinese population. We found that there were significant differences in these values between sexes, ages, races, and vendor groups, and concluded that normal 3D‐RV values should be stratified by sex, age, and vendor for clinical applications.

## 2 RESULTS

### 2.1 Demographic characteristics of the study population

A total of 1117 healthy volunteers were enrolled from 28 collaborating laboratories in 20 provinces of China. 3D‐RV images with an average frame rate of 31 Hz from 747 volunteers whose image quality was optimal without missing data, stitch artifacts, and dropouts were ultimately analyzed via Tomtec software (Figure S1 and Table S1). There were 340 men, aged 46.9 ± 16.3 years, and 407 women, aged 46.5 ± 16.2 years. The demographic features of these subjects are shown in Table 1. The median age of the entire study population was 46 years (range 19−80 years). Using Yangtze River as the boundary,^15^ China can be divided into northern and southern China, and there were not significant age and sex differences of the study population between the northern and southern China (Table S2).

**TABLE 1:** Demographic characteristics of the study population stratified by sex and age.

	Men				Women			
Parameters	Total (*n* = 340)	18‒40 (*n* = 141)	41‒65 (*n* = 136)	>65 (*n* = 63)	Total (*n* = 407)	18‒40 (*n* = 163)	41‒65 (*n* = 177)	>65 (*n* = 67)
Age (year)	46.9 ± 16.3	30.9 ± 4.9	52.2 ± 7.1^a^	71.8 ± 3.8^a^, ^b^	46.5 ± 16.2	29.6 ± 4.8	52.5 ± 6.9^a^	71.2 ± 3.9^a^, ^b^
Height (cm)	171.5 ± 5.9	173.1 ± 5.9	170.6 ± 5.2^a^	169.8 ± 6.4^a^	160.3 ± 5.5^c^	162.3 ± 5.2^c^	159.6 ± 5.0^a^, ^c^	156.9 ± 5.6^a^, ^b^, ^c^
Weight (kg)	67.9 ± 7.8	68.8 ± 8.1	68.0 ± 7.0	65.5 ± 8.3^a^, ^b^	55.9 ± 6.5^c^	55.3 ± 5.9^c^	56.9 ± 6.6^a^, ^c^	54.5 ± 7.0^b^, ^c^
BMI (kg/m^2^)	23.0 ± 2.0	22.9 ± 2.0	23.3 ± 1.9	22.5 ± 1.9^b^	21.7 ± 2.2^c^	21.0 ± 1.8^c^	22.3 ± 2.2^a^, ^c^	22.1 ± 2.4^a^
BSA (m^2^)	1.79 ± 0.1	1.81 ± 0.1	1.79 ± 0.1	1.76 ± 0.1	1.58 ± 0.1^c^	1.58 ± 0.1^c^	1.59 ± 0.1^c^	1.54 ± 0.1^a^, ^b^, ^c^
SBP (mmHg)	122.9 ± 10.1	121.4 ± 8.9	123.4 ± 11.0	125.9 ± 10.3^a^	117.3 ± 11.8^c^	111.7 ± 9.0^c^	119.3 ± 11.7^a^, ^c^	124.8 ± 12.2^a^, ^b^
DBP (mmHg)	76.9 ± 7.1	76.1 ± 6.8	77.6 ± 7.5	77.1 ± 6.7	73.0 ± 7.8^c^	70.7 ± 6.8^c^	74.3 ± 8.0^a^, ^c^	74.9 ± 8.1^a^
HR (beats/min)	71.0 ± 8.7	72.8 ± 9.5	69.6 ± 7.6^a^	69.8 ± 8.7	72.2 ± 9.2	74.9 ± 10.3	70.5 ± 8.2^a^	70.5 ± 7.4^a^

*Note*: Data are expressed as mean ± standard deviation.

Abbreviations: BMI, body mass index; BSA, body surface area; DBP, diastolic blood pressure; HR, heart rate; SBP, systolic blood pressure.

^a^ *p* < 0.05 versus subjects aged 18‒40 years in the same sex group.

^b^ *p* < 0.05 versus subjects aged 41‒65 years in the same sex group.

^c^ *p* < 0.05 versus men in the whole population or men in the same age group.

### 2.2 Sex differences in 3D‐RV volume and function

As listed in Table 2, 3D‐RV images were analyzed by Tomtec software and the normal reference values for right ventricular end‐diastolic volume (RVEDV), right ventricular end‐systolic volume (RVESV), right ventricular stroke volume (RVSV), and right ventricular ejection fraction (RVEF) were obtained from the entire study population. The RVEDV and RVESV were significantly larger in men than in women (*p* < 0.05), even after indexing to body surface area (BSA). The RVSV was significantly larger in men than in women (*p* < 0.05), and the difference disappeared after indexing to BSA. In contrast, the RVEF was higher in women than in men (*p* < 0.05).

**TABLE 2:** Three‐dimensional‐right ventricular volume and function measurements in the study population and in men and women.

Parameters	Total (*n* = 747)	Men (*n* = 340)	Women (*n* = 407)
3D‐RVEDV (mL)	82.7 ± 21.3	90.7 ± 22.2	76.0 ± 18.0^a^
3D‐RVEDVi (mL/m^2^)	49.3 ± 11.0	50.6 ± 11.5	48.2 ± 10.4^a^
3D‐RVESV (mL)	37.3 ± 11.3	41.5 ± 11.7	33.8 ± 9.6^a^
3D‐RVESVi (mL/m^2^)	22.2 ± 5.9	23.1 ± 6.1	21.4 ± 5.7^a^
3D‐RVSV (mL)	45.4 ± 11.6	49.3 ± 12.3	42.2 ± 9.9^a^
3D‐RVSVi (mL/m^2^)	27.2 ± 6.2	27.4 ± 6.4	26.9 ± 6.0
3D‐RVEF (%)	55.2 ± 5.0	54.5 ± 4.7	55.9 ± 5.0^a^

*Note*: Data are analyzed with the Tomtec software and expressed as mean ± standard deviation.

Abbreviations: 3D‐RVEDV, 3D‐right ventricular end‐diastolic volume; 3D‐RVEDVi, 3D‐right ventricular end‐diastolic volume index; 3D‐RVEF, 3D‐right ventricular ejection fraction; 3D‐RVESV, 3D‐right ventricular end‐systolic volume; 3D‐RVESVi, 3D‐right ventricular end‐systolic volume index; 3D‐RVSV, 3D‐right ventricular stroke volume; 3D‐RVSVi, 3D‐right ventricular stroke volume index.

^a^ *p* < 0.05 versus men.

### 2.3 Age dependency of 3D‐RV volume and function

As shown in Table 3, the mean values of RVEDV gradually decreased with age in men and women (*p* < 0.05), even after indexing to BSA. The young and middle age groups had larger RVEDV and right ventricular end‐diastolic volume index (RVEDVi) than did the old age group in both men and women (*p* < 0.05). Similarly, the mean values of RVESV gradually decreased with age in men (*p* < 0.05), but this difference disappeared after indexing to BSA. In contrast, the mean values of RVESV and right ventricular end‐systolic volume index (RVESVi) did not vary with age in women (*p* > 0.05). The mean values of RVSV gradually decreased with age in men and women (*p* < 0.05), even after indexing to BSA. The mean values of RVEF gradually decreased with age in men and women (*p* < 0.05), and the young age group had higher RVEF than did the middle‐aged and older age groups (*p* < 0.05).

**TABLE 3:** Three‐dimensional‐right ventricular volume and function in men and women stratified by age.

	Men			Women		
Parameters	18‒40 (*n* = 141)	41‒65 (*n* = 136)	>65 (*n* = 63)	18‒40 (*n* = 163)	41‒65 (*n* = 177)	>65 (*n* = 67)
3D‐RVEDV (mL)	94.7 ± 21.7	90.4 ± 22.8	82.4 ± 20.0^a^, ^b^	78.7 ± 18.5	75.2 ± 18.6	71.7 ± 14.3^a^
3D‐RVEDVi (mL/m^2^)	52.3 ± 11.3	50.2 ± 11.8	47.4 ± 10.9^a^	50.0 ± 10.7	47.4 ± 10.7^c^	46.3 ± 8.1^a^
3D‐RVESV (mL)	42.7 ± 12.1	41.8 ± 11.7	38.1 ± 10.4^a^, ^b^	34.3 ± 10.1	33.8 ± 9.9	32.4 ± 7.8
3D‐RVESVi (mL/m^2^)	23.6 ± 6.4	23.1 ± 5.9	21.9 ± 5.7	21.7 ± 6.0	21.3 ± 5.8	21.0 ± 4.5
3D‐RVSV (mL)	52.0 ± 11.7	48.7 ± 12.8^c^	44.3 ± 10.8^a^, ^b^	44.4 ± 10.1	41.4 ± 9.9^c^	39.3 ± 7.8^a^
3D‐RVSVi (mL/m^2^)	28.7 ± 6.1	27.0 ± 6.6^c^	25.4 ± 6.0^a^	28.2 ± 6.2	26.2 ± 6.0^c^	25.6 ± 5.0^a^
3D‐RVEF (%)	55.2 ± 5.1	54.0 ± 4.4^c^	54.0 ± 4.6	56.7 ± 5.2	55.4 ± 4.9^c^	55.1 ± 4.6^a^

*Note*: Data are analyzed with the Tomtec software and expressed as mean ± standard deviation.

Abbreviations: 3D‐RVEDV, 3D‐right ventricular end‐diastolic volume; 3D‐RVEDVi, 3D‐right ventricular end‐diastolic volume index; 3D‐RVEF, 3D‐right ventricular ejection fraction; 3D‐RVESV, 3D‐right ventricular end‐systolic volume; 3D‐RVESVi, 3D‐right ventricular end‐systolic volume index; 3D‐RVSV, 3D‐right ventricular stroke volume; 3D‐RVSVi, 3D‐right ventricular stroke volume index.

^a^ *p* < 0.05 versus subjects aged 18−40 years.

^b^ *p* < 0.05 versus subjects aged 41−65 years.

^c^ *p* < 0.05 versus subjects aged 18−40 years.

### 2.4 Comparison of 3D‐RV volume and function with normal values recommended by guidelines

**FIGURE 1:**
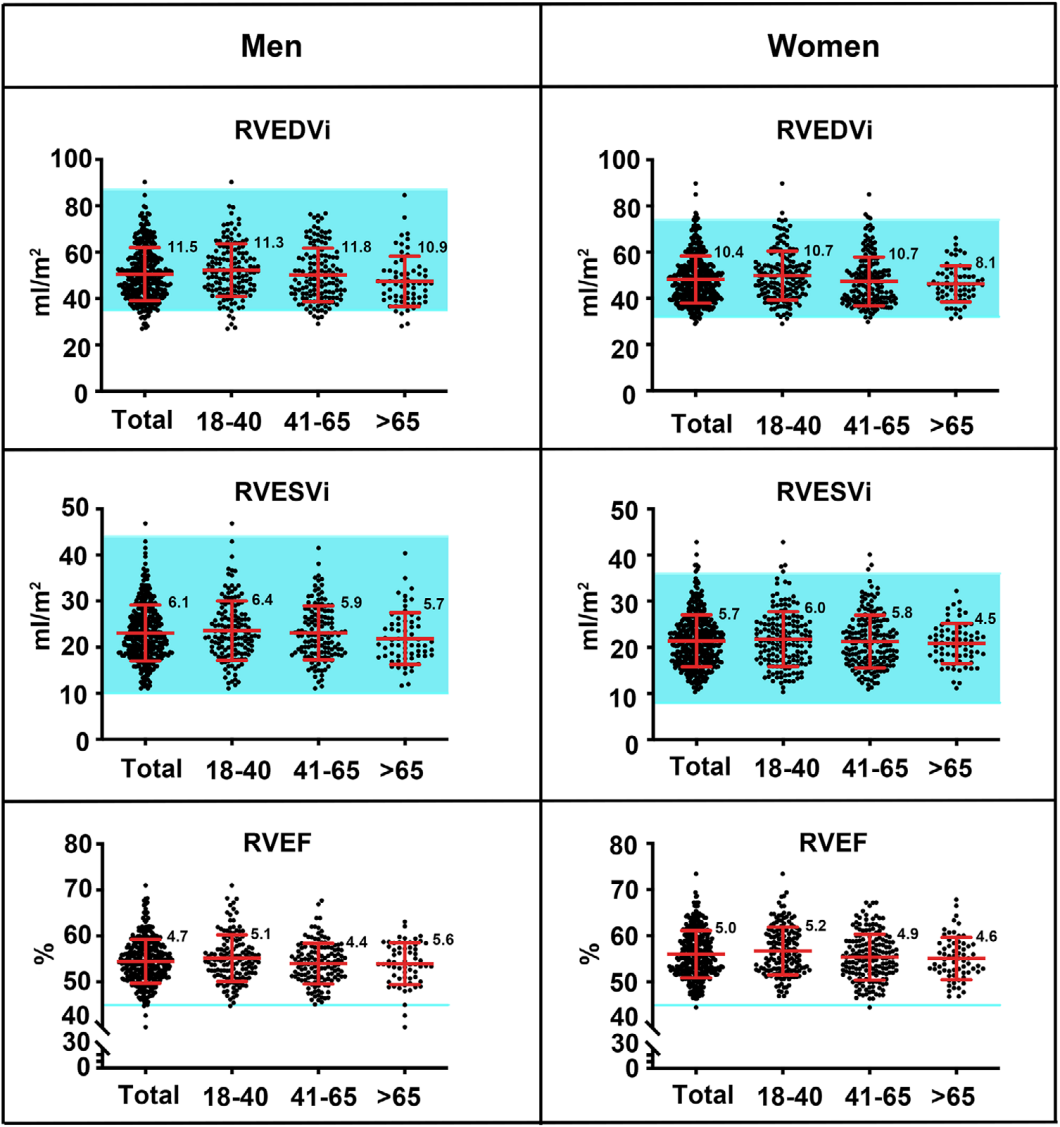
Normal values of right ventricular end‐diastolic volume index (RVEDVi), right ventricular end‐systolic volume index (RVESVi), and right ventricular ejection fraction (RVEF) stratified by sex and age in echocardiographic measurements in normal Chinese adults (EMINCA) II were compared with those recommended by the American Society of Echocardiography/European Association of Cardiovascular Imaging (ASE/EACVI) guidelines. The data are expressed as mean and standard deviation. The means and standard deviations are presented with red bars and the exact values of the standard deviation were annotated in each group. The individual values are presented as black dots. The normal ranges of RVEDVi and RVESVi recommended by the guidelines are presented by blue horizontal area and the lower normal limits of RVEF recommended by the guidelines are presented by blue horizontal lines.

The upper and lower normal limits of 3D‐RV volume and function measured by the Tomtec system in both men and women are presented in Table 4. The means and individual values of the RVEDVi, RVESVi, and RVEF measured by the Tomtec system in men and women were compared with those recommended by the 2015 ASE/EACVI guidelines based on meta‐analysis data (Figure 1). As shown in Table 4 and Figure 1, our results showed that the upper and lower normal limits of the RVEDVi measured by Tomtec software in men were lower than the upper and lower normal limits recommended by the ASE/EACVI guidelines, respectively.^12^ In contrast, the upper and lower normal limits of the RVEDVi in women were consistent with the upper and lower normal limits proposed by the ASE/EACVI guidelines. The normal ranges of the RVESVi for both men and women fell within those recommended by the ASE/EACVI guidelines. By comparison, the lower normal limit of RVEF was 45% according to the ASE/EACVI guidelines, whereas our results demonstrated that the lower normal limits of RVEF were 46% in men and 47% in women. In addition, the mean values of RVEDVi, RVESVi, and RVEF were significantly lower than the corresponding guideline values for both men and women (Table 5 and Figure S2). These results suggest that mild 3D‐RV enlargement in Chinese patients may be labeled as a normal RV size according to the ASE/EACVI guidelines. Similarly, mild 3D‐RV systolic dysfunction in Chinese patients may be regarded as normal RV function according to the ASE/EACVI guidelines, leading to misdiagnosis in these patients.

**TABLE 4:** Normal value ranges of three‐dimensional‐right ventricular volume and function in men and women.

Parameters	Men (LNL to UNL)	Women (LNL to UNL)
3D‐RVEDV (mL)	56.7‒144.5	49.9‒119.3
3D‐RVEDVi (mL/m^2^)	31.9‒75.5	33.0‒72.0
3D‐RVESV (mL)	22.9‒67.5	18.4‒56.1
3D‐RVESVi (mL/m^2^)	12.4‒36.6	12.3‒34.2
3D‐RVEF (%)	46.1‒65.5	47.2‒66.0

*Note*: Data were analyzed with the Tomtec software. Normal ranges were defined as the upper and lower limits using 2.5th and 97.5th percentiles from the corresponding group.

Abbreviations: 3D‐RVEDV, 3D‐right ventricular end‐diastolic volume; 3D‐RVEDVi, 3D‐right ventricular end‐diastolic volume index; 3D‐RVEF, 3D‐right ventricular ejection fraction; 3D‐RVESV, 3D‐right ventricular end‐systolic volume; 3D‐RVESVi, 3D‐right ventricular end‐systolic volume index; LNL, lower normal limits; UNL, upper normal limits.

**TABLE 5:** Three‐dimensional‐right ventricular volume and function measurements in echocardiographic measurements in normal Chinese adults (EMINCA) II and American Society of Echocardiography/European Association of Cardiovascular Imaging guidelines.

Parameters	EMINCA II	Guidelines	*p*‐Value
3D‐RVEDVi (mL/m^2^)			
Men	51 ± 11.5	61 ± 13	<0.001
Women	48 ± 10.4	53 ± 10.5	<0.001
3D‐RVESVi (mL/m^2^)			
Men	23 ± 6.1	27 ± 8.5	<0.001
Women	21 ± 5.7	22 ± 7	0.047
3D‐RVEF (%)	55 ± 5	58 ± 6.5	<0.001

*Note*: Data are analyzed with the Tomtec software and expressed as mean ± standard deviation.

Abbreviations: 3D‐RVEDVi, 3D‐right ventricular end‐diastolic volume index; 3D‐RVEF, 3D‐right ventricular ejection fraction; 3D‐RVESVi, 3D‐right ventricular end‐systolic volume index.

### 2.5 Comparison of 3D‐RV volume and function between vendors

A recent WASE study noted intervendor variability in 3D‐left ventricular (3D‐LV) volume and function.^16^ However, the intervendor variability of 3D‐RV volume and function has not been reported. To examine the difference in 3D‐RV volume and function measured by the two vendor systems, we divided the study population into two vendor groups: the General Electric company (GE) group, in whom 3D‐RV values were acquired by VividE9 or VividE95 (*n* = 287), and the Phillips group, in whom 3D‐RV values were acquired by IE33 or Epiq7c (*n* = 460). There was no difference in demographic features in terms of sex, age, height, or weight between the two vendor groups (Table S3); thus, the 3D‐RV measurements derived from the two groups were comparable. We conducted between‐vendor comparisons in five steps. In step 1, all 3D‐RV images acquired from the two vendor groups were analyzed by vendor‐independent software Tomtec. As shown by P1 (V1 vs. V3) in Table 6, there were no significant differences in the RVEDV, RVEDVi, or RVESVi. However, there were significant differences in the RVESV and RVEF, suggesting that there existed some differences in image acquisition between the two vendors.

**TABLE 6:** Three‐dimensional‐right ventricular measurements by two vendors’ instruments and three software platforms in the study population.

Vendors	GE (VividE9, VividE95, *n* = 287)		Phillips (IE33, Epiq7c, *n* = 460)					
Software platforms	Tomtec (V1)	Echopac (V2)	Tomtec (V3)	Qlab (V4)	P1 (V1 vs. V3)	P2 (V1 vs. V2)	P3 (V3 vs. V4)	P4 (V2 vs. V4)
3D‐RVEDV (mL)	52.1‒139.2	52.0‒127.4	50.6‒130.4	52.0‒143.7	0.836	0.042	<0.001	<0.001
3D‐RVEDVi (mL/m^2^)	33.3‒73.9	32.3‒69.2	31.9‒74.7	34.2‒78.6	0.166	0.010	<0.001	<0.001
3D‐RVESV (mL)	19.3‒67.7	21.0‒66.0	19.1‒59.2	21.3‒70.0	0.025	0.074	<0.001	<0.001
3D‐RVESVi (mL/m^2^)	12.4‒37.8	12.5‒38.2	12.3‒33.9	14.8‒38.6	0.339	0.039	<0.001	<0.001
3D‐RVEF (%)	46.4‒65.3	43.5‒65.6	47.3‒66.6	44.4‒63.6	<0.001	0.732	<0.001	0.040

*Note*: Data were expressed as the upper and lower normal limits using 2.5th and 97.5th percentiles from the corresponding group.

Abbreviations: 3D‐RVEDV, 3D‐right ventricular end‐diastolic volume; 3D‐RVEDVi, 3D‐right ventricular end‐diastolic volume index; 3D‐RVEF, 3D‐right ventricular ejection fraction; 3D‐RVESV, 3D‐right ventricular end‐systolic volume; 3D‐RVESVi, 3D‐right ventricular end‐systolic volume index.

In step 2, 3D‐RV images from the same GE group were analyzed by Tomtec and Echopac software. As shown by P2 (V1 vs. V2) in Table 6, there were significant differences in the RVEDV, RVEDVi, and RVESVi, suggesting that there were some differences in image analysis between the two software systems.

In step 3, 3D‐RV images from the same Phillips group were analyzed by Tomtec and Qlab software. As demonstrated by P3 (V3 vs. V4) in Table 6, there were significant differences in all 3D‐RV values, including the RVEDV, RVEDVi, RVESV, RVESVi, and RVEF, suggesting that there was a fundamental difference in image analysis between the two software systems.

In step 4, 3D‐RV images acquired from the two vendor groups were analyzed by the vendor‐dependent software packages Echopac and Qlab. As demonstrated by P4 (V2 vs. V4) in Table 6, there were significant differences in all 3D‐RV values, including the RVEDV, RVEDVi, RVESV, RVESVi, and RVEF, suggesting that there was a fundamental difference in image acquisition and analysis between the two vendors (*p* < 0.05).

In step 5, to further verify the difference in image acquisition between the two vendors, a separate group of 50 healthy volunteers (Table S4) were recruited and each of these volunteers underwent echocardiographic examinations twice a day performed by the same echocardiographic expert using both the VividE95 and Epiq7c systems and their image data were analyzed by the same vendor‐independent software Tomtec. To exclude the effect of poor chest window on image acquisition, younger volunteers were recruited in the separate group than in the entire study population (34.5 ± 13.9 years vs. 46.7 ± 16.2 years, *p* < 0.001). As shown in Table S5, there were still significant differences in the RVEDV, RVEDVi, and RVEF, suggesting that there were indeed some differences in image acquisition between the two vendors. These results demonstrated for the first time that there was a significant difference in 3D‐RV volume and function across the two imaging methods, which was induced not only by differences in image analysis using different software programs but also by differences in image acquisition using different ultrasound systems. Thus, image acquisition and analysis are important variables affecting normal values of 3D‐RV volume and function, apart from age, sex, and race.

### 2.6 Comparison of 3D‐RV measurements with published data

Wang et al. presented a systematic review and meta‐analysis^7^ involving 25 small studies including 2165 subjects, the majority of which were being white subjects. The values of 3D‐RV volume and 3D‐RVEF were higher in this meta‐analysis than those in the present study. These differences may be attributed to differences in different body size between the recruited populations and the different software systems used in the two studies. We also compared 3D‐RV volume and function between the EMINCA II study and several relatively large studies (*n* = 245‒300), which included mostly white individuals (Table S6).^9^, ^17^, ^18^, ^19^ All these previous studies showed larger 3D‐RV volume and 3D‐RVEF than did the EMINCA II study. In the study by Maffessanti et al.,^10^ 507 normal subjects were recruited, and only the median and 5th and 95th percentiles of the RV were reported. As shown in Table S7, the 3D‐RV volume and 3D‐RVEF in Maffessanti's study were considerably greater than those in the EMINCA II. Finally, in a recent WASE study on 3D‐RV volume and function, the white population exhibited larger values of 3D‐RV volume and function than did the Asian population.^11^

### 2.7 Comparison between 3D‐RV and 3D‐LV measurements in EMINCA II

As shown in Table S8, 684 healthy Chinese volunteers had adequate image quality for both 3D‐LV and 3D‐RV measurements in the EMINCA II; thus, in these volunteers, 3D‐RV and 3D‐LV measurements were compared. We found that in the same subjects 3D‐RV volume measurements, including end‐diastolic volume (EDV) and end‐systolic volume (ESV), were significantly larger than the corresponding 3D‐LV volume measurements, even after indexing by BSA. And the values of 3D‐LV ejection fraction (LVEF) were significantly greater than those of 3D‐RVEF. However, the values of 3D‐LVSV and 3D‐RVSV showed good agreement (46.7 mL vs. 46.0 mL), with no significant difference between the two measurements.

### 2.8 Reproducibility

As shown in Figure S3, there was good intraobserver and interobserver reproducibility in the 3D‐RV measurements. The intraclass Person correlation coefficients for intraobserver reproducibility of the RVEDV, RVESV, and RVEF were 0.983, 0.975, and 0.923, respectively (*p* < 0.001 for all). The intraclass correlation coefficients for interobserver reproducibility of the RVEDV, RVESV, and RVEF were 0.975, 0.961, and 0.899, respectively (*p* < 0.001 for all). The intraobserver reproducibility of the coefficient of variance (COV) for the RVEDV, RVESV, and RVEF was 2.79%, 3.87%, and 1.92%, respectively. The COVs for interobserver reproducibility of the RVEDV, RVESV, and RVEF were 4.09%, 2.71%, and 2.24%, respectively. Although the limits of agreement for both intraobserver and interobserver reproducibility were greater than ±10 mL for the RVEDV, this variability was much lower than that in a previous study.^20^

## 3 DISCUSSION

The EMINCA II study was a prospective, nationwide, and multicenter study that aimed to define the normal reference values of 3D‐RV volume and function in a large cohort of healthy Han volunteers over a wide range of ages. There were several important findings in this study. First, a set of normal values of 3D‐RV volume and function in a large Chinese population was first presented. Second, there were substantial differences in normal values of 3D‐RV volume and function between different sex and age groups, a finding similar to that in the previous EMINCA study,^13^ suggesting that 3D‐RV normal values should be sex and age stratified. Third, the derived normal values of 3D‐RV volume and function were significantly smaller than those recommended by the ASE/EACVI guidelines for both sexes, indicating that these normal values should be race stratified. Fourth, significant intervendor differences in 3D‐RV volume and function existed, which were induced by differences in both image acquisition and analysis, suggesting that currently, normal 3D‐RV values should also be vendor stratified and that further standardization of 3D‐RV image acquisition and analysis is warranted. Finally, contrary to the official statement of the American Thoracic Society,^3^ we found good intraobserver and interobserver reproducibility in 3D‐RV measurements using updated ultrasound and software systems. To the best of our knowledge, these findings have not been reported previously in the literature.

Previous studies have shown that RV mass, volume and function measured by CMR vary significantly with age, sex, and race.^21^, ^22^ Despite a higher reproducibility for measuring RV volume and function, CMR is more time‐consuming and expensive than echocardiography, and not suitable for patients with pace‐maker implantation, prosthetic valve replacement, and claustrophobia. In contrast, echocardiography is a relatively low‐cost and convenient method for bedside real‐time monitoring and suitable for follow‐up or serial examination. Compared with 2D echocardiography that can only image part of the right ventricle, 3D echocardiography can display the entire right ventricle allowing a more accurate assessment of RV volume and function. Available evidence has shown that RV volume and RVEF measured by 3D echocardiography correlate closely with CMR measurement.^8^ Like those of CMR, the present study demonstrated smaller 3D‐RV volume and higher RVEF in women than in men. In addition, the 3D‐RVEDV and 3D‐RVEDVi decreased with age. Although a previous study showed that older age was associated with higher RVEF (an expected increase of 1% per decade),^10^ we found only a weak negative correlation between age and RVEF in women, with a slight decrease in RVEF with age. Thus, unless an ideal indexation method is developed that can correct for the impact of sex and age on 3D‐RV volume and function, normal values of these measurements should be sex and age stratified.

A previous WASE 3D‐LV study showed that Asians had significantly smaller LV volumes than whites and blacks did, even after adjusting for isometric BSA.^23^ A recently published WASE 3D‐RV study obtained similar results.^11^ Our results showed that the upper and lower normal limits of the RVEDVi measured in men were lower than the upper and lower normal limits recommended by the ASE/EACVI guidelines, respectively. In addition, the mean values of the RVEDVi, RVESVi, and RVEF were significantly smaller than the corresponding values recommended by guidelines for both sexes. A possible explanation for these differences is the smaller body size in the Chinese population than in the Caucasian population. Furthermore, the lower normal limits of the RVEF in men (46%) and women (47%) in the present study were all greater than those recommended by the ASE/EACVI guidelines (45%).^12^ These results suggested a possible difference in 3D‐RV volume and function between different races. Thus, normal values of these measurements should also be race stratified. Based on the results in EMINCA II, we defined an enlarged 3D‐RV as RVEDVi > 76 mL/m^2^ in men and RVEDVi > 72 mL/m^2^ in women, and reduced 3D‐RV systolic function as RVEF < 46% in men and RVEF < 47% in women. By comparison of these criteria with those defined by the ASE/EACVI guidelines, we found that 14 individuals in the EMINCA II study had their RVEDVi and RVEF values falling with the normal ranges recommended by the ASE/EACVI guidelines, but outside the normal ranges defined by the EMINCA II, which suggested that in clinical practice, a considerable proportion of Chinese patients with mildly enlarged RV and reduced RVEF would be classified as healthy subjects by the ASE/EACVI guidelines. Thus, the diagnostic criteria of 3D‐RV enlargement and systolic dysfunction defined by the present study should be adopted in dealing with Chinese cardiac patients in clinical practice.

As 3D‐RV volume and function are affected by sex, age, and race, an ideal indexing method that can correct for the impact of all physiological variables on 3D‐RV measurements is highly warranted. At present, indexing echocardiographic measurements of cardiac chambers by BSA is recommended by the ASE/EACVI guidelines.^12^ However, our previous study revealed that the total success rate of correcting for 34 2D echocardiographic parameters, including isometric weight, height, body mass index, and BSA was only 11%.^24^ Similarly, the WASE 3D‐LV study failed to correct for the impact of race on 3D‐LV volume using isometric BSA.^23^ In the present study, there was still a difference in the RVEDVi and RVESVi between men and women, even after indexing by isometric BSA, indicating that indexing method recommended by current guidelines should be abandoned. Recently, Nabeshima et al. used several methods including BSA, BSA^1.5^, BSA^1.8^, height, height^2.3^, height^2.9^, and estimated lean body mass, to index of 3D‐LV end‐diastolic volume; however, these methods failed to correct the difference between races and nationalities.^25^ Recently, our group developed an optimized multivariate allometric model using different scaling equations for different echocardiographic parameters, and achieved a high success rate of correction (100%) for 34 parameters measured by 2D echocardiography.^24^ However, whether this model can be used to correct physiological variance in 3D‐RV measurements awaits further investigation.

Recent studies have shown differences in 3D‐RV volume and function measured by different ultrasound and software systems, but the underlying mechanism is unclear.^7^, ^26^ In this study, we compared 3D‐RV measurements of ultrasound systems made by two vendors: GE instruments (VividE9 and VividE95) and Phillips instruments (IE33 and EpiQ7c). In addition, we compared three software systems: two vendor‐dependent software programs, including Echopac and Qlab, and one vendor‐independent software package, Tomtec. After in‐depth data analysis, we identified several sources of differences between the two vendors: (1) differences in image acquisition between the GE and Phillips ultrasound systems; (2) differences in image analysis between the Tomtec and Echopac software systems; (3) differences in image analysis between the Tomtec and Qlab software systems; and (4) differences in image analysis between the Echopac and Qlab software systems. Thus, unlike 2D echocardiography, between‐vendor differences constitute an important variable affecting 3D RV measurements. At present, to make a correct comparison between 3D‐RV measurements in clinical practice, normal values stratified by sex, age, race, and vendor are needed.

In the present study, we compared 3D‐RV values in the current EMINCA II study and those reported in previous ones where the majority of the participants were white, and the largest sample included only 300 participants with a statistical power insufficient for age‐ and sex‐stratified analysis. Despite these limitations inherent in previous studies, we found that values of 3D‐RV volume and function were significantly greater in reported studies than those in EMINCA II. These differences may primarily result from body size differences between Chinese and Caucasian populations and, to a lesser extent, from differences in the ultrasound and software systems used in our study and previous ones. A recently published WASE study on 3D‐RV volume and function demonstrated larger 3D‐RV volume and function in whites than in Asian population, lending support to the results of the EMINCA II.^11^

In the current study, 3D‐RV and 3D‐LV measurements were compared in the same study population in the EMINCA II with optimal images of both the right and left ventricles. Values of 3D‐EDV and 3D‐ESV were significantly larger in the right ventricle than in the left ventricle, and values of 3D‐LVEF were greater than 3D‐RVEF, possibly due to anatomical and functional differences between the two ventricles. However, the SV values were similar between the right and left ventricles, demonstrating the continuity of blood flow through the systemic and pulmonary circulations.

There were a few limitations to our study. Only Han Chinese volunteers were recruited for this study, and the results obtained may not be applicable to other ethnical populations. In addition, the feasibility of measuring 3D‐RV volume and function was only 67% (747 out of 1117 subjects) in EMINCA II, while the same feasibility was only 52% in the WASE study,^11^ indicating that in 33%‒48% of recruited subjects, RV volume and function cannot be assessed by current 3D technology. Finally, the limits of agreement for both intraobserver and interobserver reproducibility were greater than ±10 mL for the RVEDV, which may originate from biases in manually marked points, axes, and endocardial surfaces, and further development in automatic marking may reduce such a variability.

## 4 CONCLUSION

A set of normal 3D‐RV volume and function values was measured in a large Chinese population via the EMINCA II. Substantial differences in normal 3D‐RV volume and function were found between the different sex and age groups, and the derived normal values were significantly smaller than those recommended by the ASE/EACVI guidelines for both sexes. Notably, significant differences in the 3D‐RV were observed between the two vendor groups, as determined by differences in image acquisition and analysis. Thus, normal 3D‐RV values should be stratified by sex, age, race, and vendor. Further standardization of 3D‐RV image acquisition and analysis techniques is warranted.

## 5 MATERIALS AND METHODS

### 5.1 Study design and population

The EMINCA II study enrolled 1117 healthy Han Chinese volunteers from 28 collaborating laboratories in 20 provinces of China. Healthy volunteers who met the inclusion and exclusion criteria were recruited from hospital staff members, health examination centers, and adjacent communities (Table S9). BSA was calculated by using the formula by Du Bois and Du Bois.^27^ The study protocol was approved by the ethics committees of all the collaborating laboratories, and informed consent was obtained from all the recruited volunteers.

### 5.2 Echocardiographic image acquisition

The medical history, physical examination data, and laboratory assay results of all volunteers were reviewed by the Organizing Committee of the EMINCA II study to determine if they met the inclusion and exclusion criteria. Transthoracic 3D echocardiography was performed in accordance with a standardized acquisition protocol recommended by ASE.^12^ One or two experienced echocardiographic experts were selected from each of the participating laboratories who had passed the national examination and received the Certification for Ultrasound in Medicine issued by the Chinese National Health Commission. All echocardiographic experts were asked to attend an intensive training course at the core laboratory (Qilu Hospital of Shandong University) to become acquainted with the study protocol and the technical requirements for echocardiographic image acquisition and measurements.

All volunteers were connected to an electrocardiograph and scanned in the left lateral decubitus position to obtain echocardiographic images.^28^ A wide‐angle, matrix‐array transducer X5‐1 probe (Phillips iE33 or EpiQ7c; Phillips Ultrasound) and 4D probe (GE VividE9 or E95; GE Vingmed Ultrasound AS) were used to cover the entire right ventricle, and a right ventricle‐focused apical four‐chamber view was acquired, which showed the largest RV basal diameter and the LV apex at the center of the sector, according to the 2015 ASE/EACVI guidelines.^12^ To minimize the impact of respiratory motion on echocardiographic parameters, RV images were recorded while the patients held their breath at the end of expiration. Before image acquisition, we used multi‐beats mode and adjusted the number of multi‐beats to four beats in both the GE and Phillips systems. The multi‐slices mode was used to avoid dropout; this mode included 12 slices of four‐, two‐, and three‐chamber views and nine circumferential views. The images were optimized by adjusting the gain, sector width, and frame rate.^28^ For the Phillips systems, the frame rate was set at no less than 1/3 heart beat for a given subject. For the GE systems, the frame rate was set at no less than 1/3 heart beats for a given subject. Four cardiac cycles were recorded and then stored in digital DICOM format.

### 5.3 Echocardiographic image analysis

**FIGURE 2:**
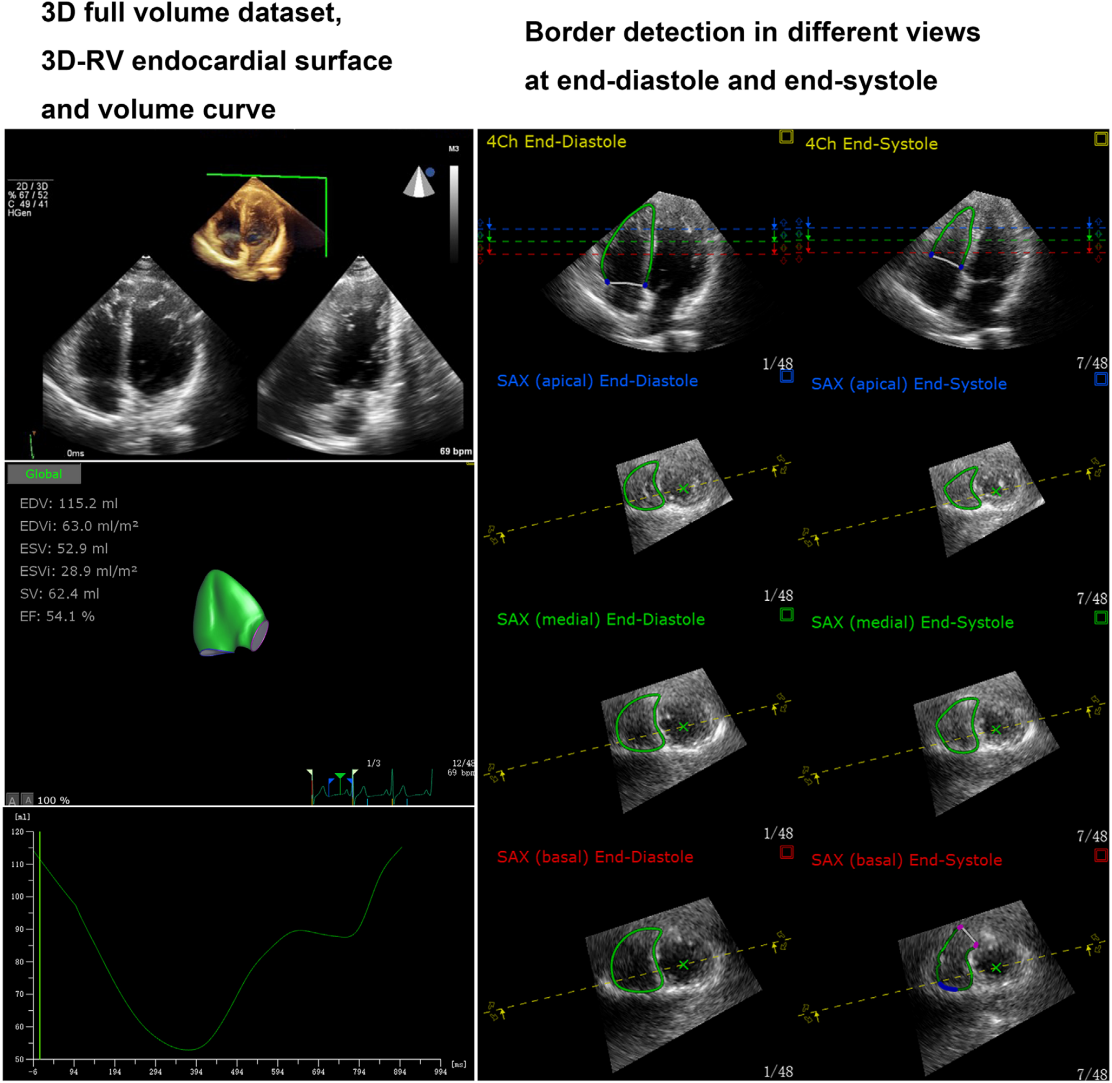
Three‐dimensional‐right ventricular (3D‐RV) image analysis was performed with the vendor‐independent software Tomtec. Left panel: 3D images of the RV‐focused apical four‐chamber view, the reconstructed 3D‐RV model and the RV volume‒time curve are displayed. Right panel: different slices of the 3D‐RV model at end‐diastole and end‐systole are shown.

Echocardiographic experts in the core laboratory and the participating laboratories reviewed the 3D‐RV images of volunteers enrolled by each participating laboratory and all stored 3D echocardiographic images were transmitted to a core laboratory at Qilu Hospital of Shandong University for analysis. 3D‐RV images were measured by two experienced echocardiographic experts (Y.Z. and Y.‐B.W) of the core laboratory in a blinded way to avoid measurement bias. Tomtec software (Tomtec Arena TTA2.31.00; TOMTEC Imaging Systems) were first used to analyze RV images of the entire study population recorded by both GE and Phillips systems according to the manufacturer's protocol^30^: (1) a series of LV and RV cross‐sections were automatically created by Tomtec software from a full‐volume dataset; (2) the LV long axis was marked as the distance between the cardiac apex and the mitral annular midpoint in the LV four‐ and two‐chamber views, respectively; (3) the RV long axis was manually marked as the distance between the cardiac apex and the tricuspid annular midpoint in the RV four‐ and two‐chamber views, respectively; (4) the diameter of the aortic annulus was manually marked in the LV three chamber view; (5) the RV short axis and the anterior and posterior junctions between RV free wall and ventricular septum were manually marked in the LV short axis view at the papillary level; (6) Tomtec system automatically traced the RV endocardial surface and generated 3D model of the right ventricle using speckle‐tracking technology, which can be manually modified with the dynamic plane tracking technique to better track RV endothelium; and (7) RVEDV, RVESV, and RVEF were calculated from the generated 3D model of the right ventricle (Figure 2).

### 5.4 Statistical analysis

The data in each subgroup are presented as the mean ± standard deviation. The lower and upper limits of the normal reference values are presented as the 2.5th and 97.5th percentiles, respectively, of the measured values from the corresponding subgroup to ensure that the derived normal values will cover 95% of the normal population. The Kolmogorov‒Smirnov test was used to explore the normality of the data distribution, and if the data distribution was normal, a *t*‐test was applied to examine the between‐group differences. If the data were not normally distributed, nonparametric statistical methods such as the Mann‒Whitney *U*‐test, were used to test for between‐group differences. Two‐tailed *p*‐values < 0.05 were considered to indicate statistical significance.

## AUTHOR CONTRIBUTIONS

M.Z. and Y.Z. were the overall principal investigators who conceived the study and obtained financial support. Y.Z. and Y.‐B.W. contributed to acquisition of data, including demographic information and 3D‐RV image of volunteers, analysis of 3D‐RV image by different software, statistical analysis, and interpretation of the results. G.‐H.Y., H.T., L.X.C., L.X.Y., T.‐G.Z., J.‐J.Y., W.H., J.Y., X.‐H.S., Y.Y., Y.‐L.W., Y.‐L.G., W.‐D.R., D.‐M.G., G.‐L.L., J.W., H.‐N.Y., Y.‐M.M., J.‐W.T., L.‐J.Y., X.‐J.M., H.‐Y.D., Y.‐C.D., M.‐Y.D., Q.Z., H.W., and D.X. contributed to acquisition of data, including demographic information and 3D‐RV image of volunteers. Y.Z. and Y.Z. contributed to writing, review, or revision of the manuscript. All authors have read and approved the final manuscript.

## CONFLICT OF INTEREST STATEMENT

The authors declare they have no conflicts of interest.

## ETHICS STATEMENT

This study was registered as ChiCTR‐OCS‐12002119 at Chinese Clinical Trial Registry (http://www.chictr.org.cn). The study protocol was approved by the Ethics Committee of Qilu Hospital of Shandong University (approval number: 2016035), which was further approved by the Ethics Committee of each participating hospital. Written informed consent was obtained from all enrolled subjects in this study.

## Supporting information

Supporting Information

## Data Availability

The data of this study are available in the article and online Supporting Information.
