# Supplementary material for: Right ventricular volume and function by three‐dimensional echocardiography: results of the echocardiographic measurements in normal Chinese adults (EMINCA) II
Source: MedComm (2020). 2024 Apr 21;5(5):e550. doi: 10.1002/mco2.550 (PMC11032740; doi:10.1002/mco2.550)
Supplement: Supplementary file 1 — Supporting Information [file MCO2-5-e550-s001.docx]

**Supplementary materials**

**Right ventricular volume and function by three-dimensional echocardiography: Results of the Echocardiographic Measurements in Normal Chinese Adults (EMINCA) II**

***By:***

Yu Zhang^1†^, Ying-Bin Wang^1†^, Gui-Hua Yao^1,2^, Hong Tang^3^, Li-Xin Chen^4^, Li-Xue Yin^5^, Tian-Gang Zhu^6^, Jian-Jun Yuan^7^, Wei Han^8^, Jun Yang^9^, Xian-Hong Shu^10^, Ya Yang^11^, Yu-Lin Wei^12^, Yan-Li Guo^13^, Wei-Dong Ren^14^, Dong-Mei Gao^15^, Gui-Lin Lu^16^, Ji Wu^17^, Hong-Ning Yin^18^, Yu-Ming Mu^19^, Jia-Wei Tian^20^, Li-Jun Yuan^21^, Xiao-Jing Ma^22^, Hong-Yan Dai^23^, Yun-Chuan Ding^24^, Ming-Yan Ding^25^, Qing Zhou^26^, Hao Wang^27^, Di Xu^28^, Mei Zhang^1^*, Yun Zhang^1^*

***From:***

^1^State Key Laboratory for Innovation and Transformation of Luobing Theory; Key Laboratory of Cardiovascular Remodeling and Function Research, Chinese Ministry of Education, Chinese National Health Commission and Chinese Academy of Medical Sciences; Department of Cardiology, Qilu Hospital of Shandong University, Jinan, China; ^2^Department of Cardiology, Qilu Hospital of Shandong University (Qingdao), Qingdao China; ^3^Department of Ultrasonography, West China Hospital, Sichuan University, Chengdu China; ^4^Department of Ultrasonography, Shenzhen People’s Hospital/The Second Clinical Medical College of Jinan University, Shenzhen China; ^5^Department of Ultrasonography, Electronic Science and Technology University of China, The Affiliated Sichuan Provincial People’s Hospital, Chengdu China; ^6^Department of Cardiology, Peking University People’s Hospital, Beijing China; ^7^Department of Ultrasonography, Henan Provincial People’s Hospital, Zhengzhou China; ^8^Department of Cardiology, The First Affiliated Hospital of Harbin Medical University, Harbin China; ^9^Department of Echocardiography, The First Affiliated Hospital of China Medical University, Shenyang China; ^10^Department of Echocardiography, Zhongshan Hospital, Fudan University, Shanghai China; ^11^Department of Echocardiography, Beijing Anzhen Hospital, Capital Medical University, Beijing China; ^12^Department of Cardiology, Sun Yat-sen Memorial Hospital, Sun Yat-sen University, Guangzhou China; ^13^Department of Ultrasonography, The Southwest Hospital of AMU, Chongqing China; ^14^Department of Ultrasonography, Shengjing Hospital of China Medical University, Shenyang China; ^15^Department of Ultrasonography, China-Japan Union hospital of Jilin University, Changchun China; ^16^Department of Ultrasonography, First Affiliated Hospital, School of Medicine, Shihezi University, Shihezi China; ^17^Department of Ultrasonography, The First Affiliated Hospital of Guangxi Medical University, Nanning China; ^18^Department of Echocardiography, The Second Hospital of Hebei Medical University, Shijiazhuang China; ^19^Department of Ultrasonography, The First Affiliated Hospital of Xinjiang Medical University, Urumqi China; ^20^Department of Ultrasonography, The Second Affiliated Hospital of Harbin Medical University, Harbin China; ^21^Department of Ultrasonography, Tangdu Hospital of Air Force Medical University of PLA, Xi’an China; ^22^Department of Ultrasonography, Wuhan Asia Heart Hospital, Wuhan China; ^23^Department of Cardiology, Qingdao Municipal Hospital, Qingdao China; ^24^Department of Ultrasonography, Yan’an Hospital Affiliated to Kunming Medical University, Kunming China; ^25^Department of Ultrasonography, The People’s Hospital of Liaoning Province, Shenyang China; ^26^Department of Ultrasonography, Renmin Hospital of Wuhan University/ Hubei General Hospital, Wuhan China; ^27^Department of Ultrasonography, Fuwai Hospital/Chinese Academy of Medical Sciences, Beijing China; ^28^Department of Ultrasonography, Jiangsu Province Hospital, Nanjing China

^†^ Yu Zhang and Ying-Bin Wang contributed equally to this work.

****Corresponding authors:*** Mei Zhang, MD, PhD, FACC, Department of Cardiology, Qilu Hospital of Shandong University, 107 Wenhuaxi Road, 250012, Jinan, China, E-mail: daixh@vip.sina.com, or Yun Zhang, MD, PhD, FACC, Hon. FASE, FESC, Department of Cardiology, Qilu Hospital of Shandong University, 107 Wenhuaxi Road, 250012, Jinan, China, E-mail: [zhangyun@sdu.edu.cn](mailto:zhangyun@sdu.edu.cn)

**Materials and methods**

**Study design and population**

To ensure an even distribution of ages in enrolled volunteers, each of the 28 collaborating laboratories was asked to enroll at least 6 healthy volunteers in each of the 6 age decade groups: 18-29 years, 30-39 years, 40-49 years, 50-59 years, 60-69 years, and >70 years, in whom men and women should account for 50%. As the age spread was wide, to simplify data analysis and presentation, both enrolled men and women were divided into three age groups following the WASE study: young age, 18-40 years, middle age, 41-65 years and old age, >65 years.^1^ The RVEDV and RVESV were also indexed to BSA.

**Echocardiographic image analysis**

In order to assess the differences in 3D-RV volume and function measured by commercially available systems, we also analyzed the images acquired by GE and Phillips systems for vendor-specific software, Echopac (Echopac version 204.0.00; GE Vingmed Ultrasound AS, GE Medical Systems) and QLAB (QLAB 13; Philips Andover, MA, USA), respectively.

**Intraobserver and interobserver variability**

To test the reproducibility of measurements, three key parameters, RVEDV, RVESV and RVEF were re-measured in 60 randomly selected subjects. Intraobserver variability was assessed by Y.Z., from Qilu Hospital of Shandong University at a four-weeks interval, and interobserver variability was assessed by Y.Z. and Y.-B.W. from Qilu Hospital of Shandong University in a blinded way. Coefficient of variance (COV) was calculated according to the formula: COV%=|x-y|/mean (x,y)×100%.

**References**

1. Asch FM, Miyoshi T, Addetia K, et al. Similarities and differences in left ventricular size and function among races and nationalities: results of the World Alliance Societies of Echocardiography Normal Values Study. *J Am Soc Echocardiogr*. 2019; 32(11):1396-1406.e2.

**Supplementary Figures**


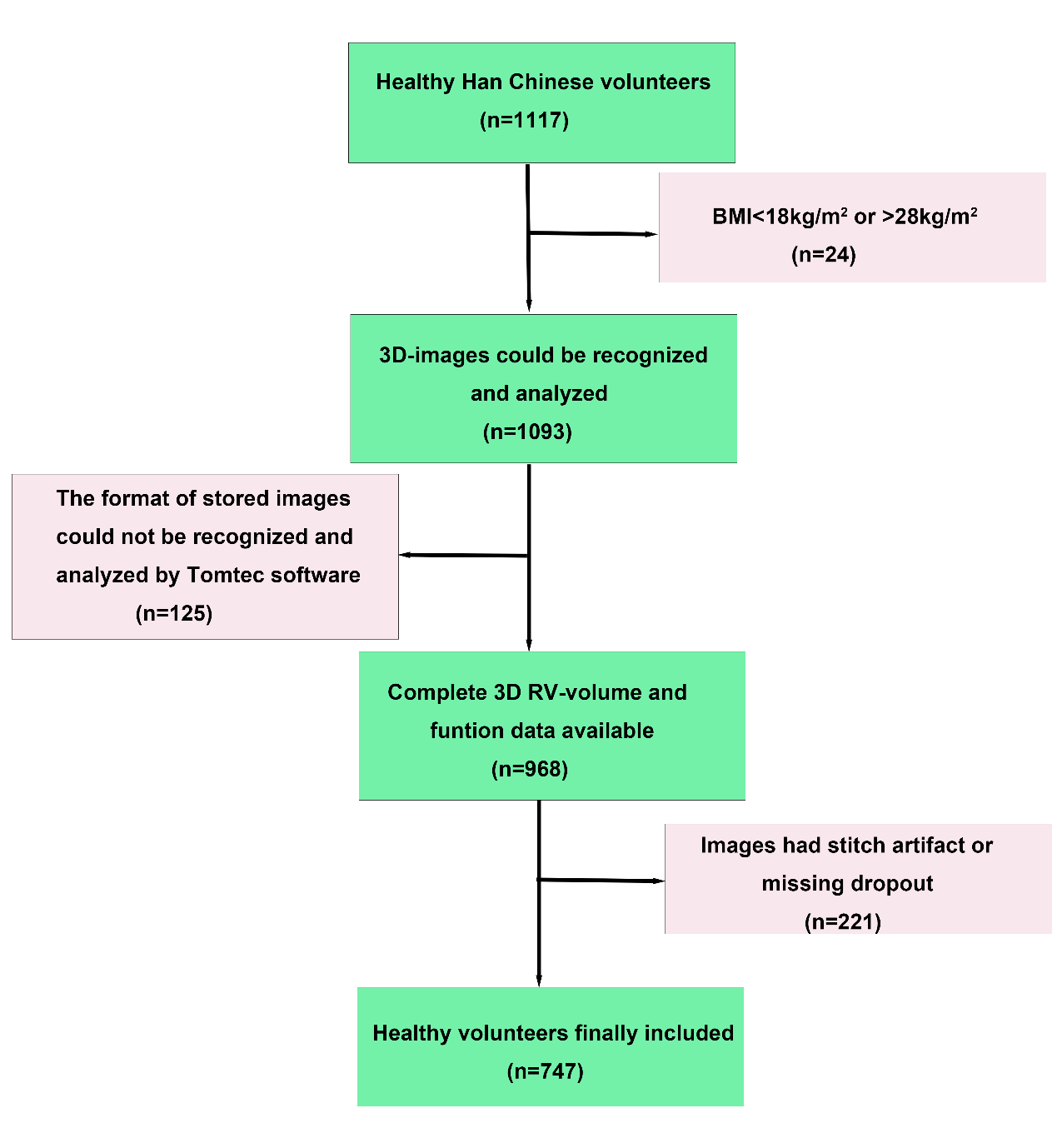


**Supplementary Figure1.** Flow diagram of the study population selection in the EMINCA II study.


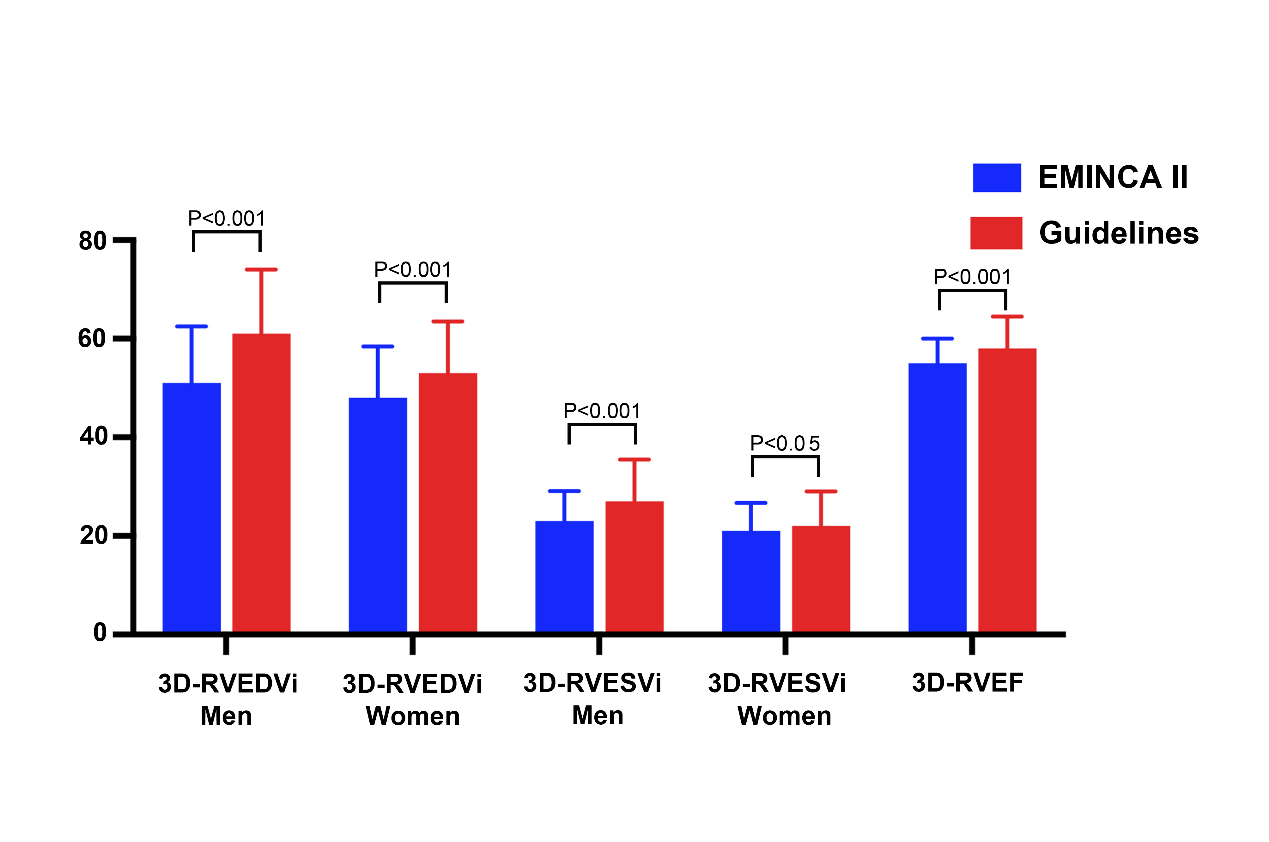


**Supplementary Figure2.** Comparison of 3D-RV measurements between EMINCA II and ASE/EACVI guidelines. The values of RVEDVi, RVESVi and RVEF in EMINCA II were significantly lower than those in ASE/EACVI guidelines for both men and women (*P<0.05*).


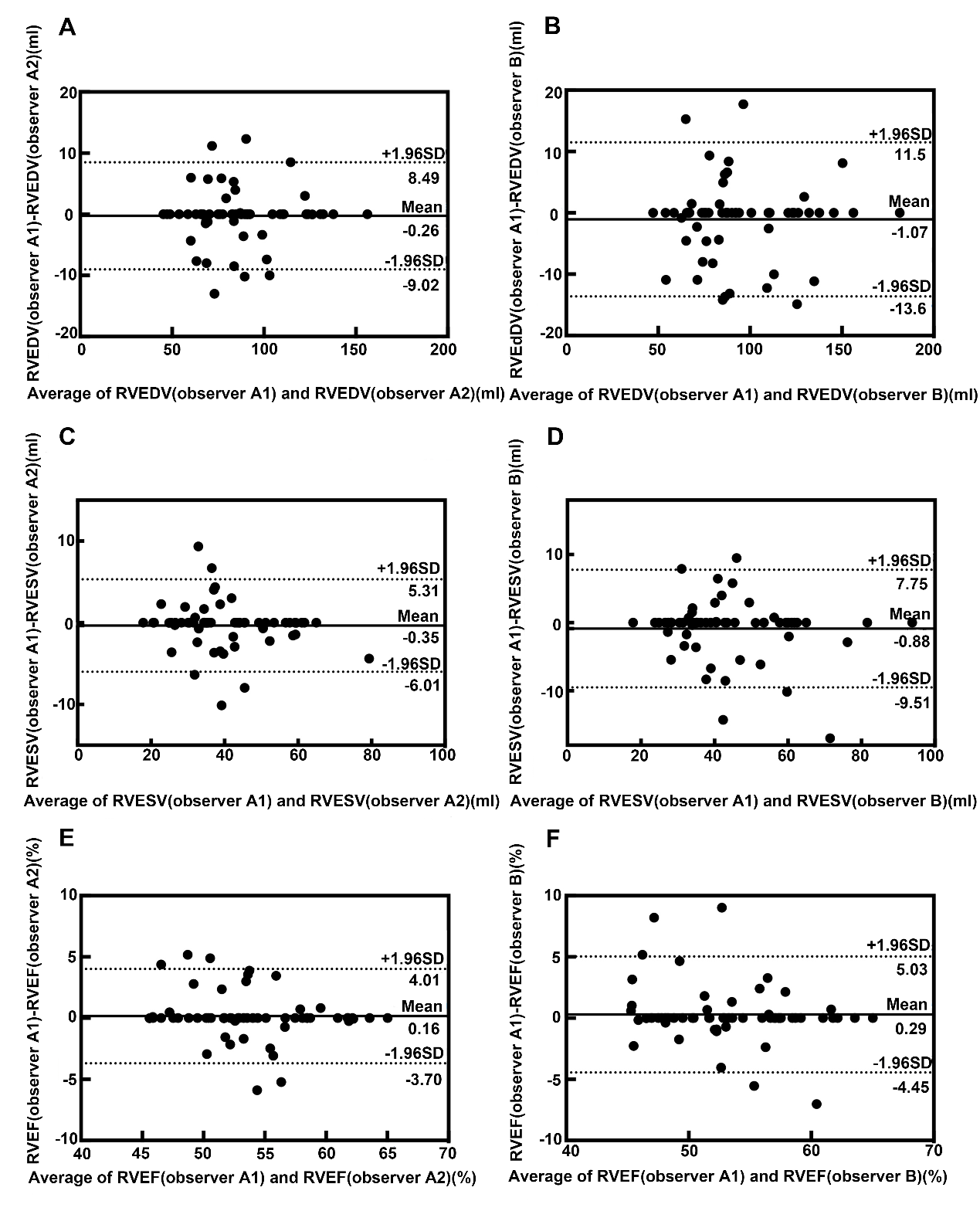


**Supplementary Figure3.** Bland-Altman plots of intraobserver and interobserver variability of the RVEDV(**A,B**), RVESV(**C,D**) and RVEF(**E,F**). RVEDV, right ventricular end-diastolic volume; RVESV, right ventricular end-systolic volume; RVEF, right ventricular ejection fraction.

**Supplementary Tables**

| **Supplementary Table 1** Volunteers enrolled from each collaborative hospital | | | |  |  |
| --- | --- | --- | --- | --- | --- |
| **Collaborative hospitals** | **Contributing**  **authors** | **Initially enrolled volunteers** | **Finally enrolled volunteers** | **Age**  **Median**  **(min, max)** | **Sex**  **Men**  **(percentage)** |
| Qilu Hospital of Shandong University（Jinan） | Yu Zhang, Ying-Bin Wang  Mei Zhang, Yun zhang | 103 | 86 | 41(22,72) | 31(36%) |
| Qilu Hospital of Shandong University (Qingdao) | Gui-Hua Yao | 86 | 57 | 42(22,80) | 25(43.8%) |
| West China Hospital, Sichuan University | Hong Tang | 65 | 59 | 54(22,80) | 28(47.5%) |
| The Second Clinical Medical College of Jinan University | Li-Xin Chen | 50 | 35 | 47(24,77) | 19(54.3%) |
| The Affiliated Sichuan Provincial People’s Hospital | Li-Xue Yin | 44 | 35 | 42(22,76) | 15(42.9%) |
| Peking University People’s Hospital | Tian-Gang Zhu | 40 | 32 | 31(22,67) | 17(53.1%) |
| Henan Provincial People’s Hospital | Jian-Jun Yuan | 41 | 28 | 46(25,79) | 12(42.9%) |
| The First Affiliated Hospital of Harbin Medical University | Wei Han | 39 | 32 | 50(26,73) | 13(40.6%) |
| The First Affiliated Hospital of China Medical University | Jun Yang | 39 | 30 | 46(23,79) | 14(46.7%) |
| Zhongshan Hospital, Fudan University | Xian-Hong Shu | 36 | 22 | 47(23,74) | 11(50%) |
| Beijing Anzhen Hospital, Capital Medical University | Ya Yang | 35 | 23 | 38(25,60) | 9(39.1%) |
| Sun Yat-sen Memorial Hospital, Sun Yat-sen University | Yu-Lin Wei | 36 | 24 | 49(21,77) | 12(50%) |
| The Southwest Hospital of AMU | Yan-Li Guo | 35 | 26 | 47(20,77) | 12(46.2%) |
| Shengjing Hospital of China Medical University | Wei-Dong Ren | 39 | 24 | 51(25,80) | 13(54.2%) |
| China-Japan Union hospital of Jilin University | Dong-Mei Gao | 41 | 24 | 46(25,77) | 13(54.2%) |
| First Affiliated Hospital, Shihezi University | Gui-Lin Lu | 30 | 24 | 50(27,74) | 12(50%) |
| The First Affiliated Hospital of Guangxi Medical University | Ji Wu | 30 | 24 | 50(23,78) | 12(50%) |
| The Second Hospital of Hebei Medical University | Hong-Ning Yin | 27 | 18 | 40(22,64) | 8(44.4%) |
| The First Affiliated Hospital of Xinjiang Medical University | Yu-Ming Mu | 26 | 17 | 50(26,77) | 10(58.8%) |
| The Second Affiliated Hospital of Harbin Medical University | Jia-Wei Tian | 24 | 13 | 41(25,60) | 5(38.5%) |
| Tangdu Hospital of Air Force Medical University of PLA | Li-Jun Yuan | 45 | 24 | 51(23,75) | 12(50%) |
| Wuhan Asia Heart Hospital | Xiao-Jing Ma | 30 | 10 | 47(19,73) | 5(50%) |
| Qingdao Municipal Hospital | Hong-Yan Dai | 30 | 15 | 36(25,78) | 7 (46.7%) |
| Yan’an Hospital Affiliated to Kunming Medical University | Yun-Chuan Ding | 24 | 22 | 49(24,76) | 11(50%) |
| The People’s Hospital of Liaoning Province | Ming-Yan Ding | 50 | 23 | 54(27,79) | 5(21.7%) |
| Renmin Hospital of Wuhan University | Qing Zhou | 24 | 15 | 50(23,75) | 7(46.7%) |
| Fuwai Hospita, Chinese Academy of Medical Sciences | Hao Wang | 24 | 3 | 51(45,60) | 2(66.7%) |
| Jiangsu Province Hospital | Di Xu | 24 | 2 | 30(26,33) | 1(50%) |

The data of age are expressed as the median age and the minimum and maximum ages in recruited volunteers in each collaborative hospital. The data of sex are expressed as the number and percentage of men in recruited volunteers in each collaborative hospital.

| **Supplementary Table 2** Comparison of age and sex between study populations recruited from northern and southern China | | | |
| --- | --- | --- | --- |
|  | Northern China (n=473) | Southern China (n=274) | P value |
| Sex |  |  |  |
| Men (percentage) | 208 (44%) | 132 (48%) | 0.267 |
| Women (percentage) | 265 (56%) | 142 (52%) |  |
| Age |  |  |  |
| Median (2.5^th^, 97.5^th^) | 44 (23, 76) | 48 (22, 76) | 0.05 |

Values are expressed as number and percentage; Continuous variables are expressed as median and 2.5 and 97.5 percentiles as they are not in normal distribution.

| **Supplementary Table 3** Demographic characteristics of volunteers in GE and Phillips groups | | | |
| --- | --- | --- | --- |
| Vendor | GE (VividE9, VividE95, n=287) | Phillips (IE33, Epiq7c, n=460) | P value |
| Sex |  |  | 0.509 |
| Men | 135(47.0%) | 204(44.3%) |  |
| Women | 152(53.0%) | 256(55.7%) |  |
| Age (years) | 46(23-76) | 46(23-76) | 0.788 |
| Height (cm) | 165(150-182) | 165(150-180) | 0.546 |
| Weight (kg) | 61.0(46.5-81.0) | 60.0(45.0-80.0) | 0.064 |

Values are expressed as number and percentage; Continuous variables are expressed as median and 2.5 and 97.5 percentiles as they are not in normal distribution.

| **Supplementary Table 4** Demographic characteristics of volunteers in a separate group | |
| --- | --- |
| Variables | Values (percentage) |
| Sex |  |
| Men | 16(32.0%) |
| Women | 34(68.0%) |
| Age (years) | 34.5±13.9 |
| Height (cm) | 166.8±7.4 |
| Weight (kg) | 60.2±9.2 |

| **Supplementary Table 5** 3D-RV images acquired by both vendors’ instruments and analyzed by Tomtec in a separate group of volunteers | | | |
| --- | --- | --- | --- |
| Vendors | GE (VividE95, n=50) | Phillips (Epiq7c, n=50) | P value |
| 3D-RVEDV (mL) | 78.7±22.7 | 90.7±29.6 | <0.001 |
| 3D-RVEDVi (mL/m^2^) | 46.8±11.0 | 53.9±14.7 | <0.001 |
| 3D-RVESV (mL) | 39.3±13.3 | 41.8±16.4 | 0.140 |
| 3D-RVESVi (mL/m^2^) | 23.3±6.6 | 24.8±8.3 | 0.134 |
| 3D-RVEF (%) | 50.6±4.5 | 54.3±6.1 | <0.001 |

Data are expressed as mean ± SD.

| **Supplementary Table 6** Comparison of 3D-RV volume and function in EMINCA II and published data | | | | | | | | | |
| --- | --- | --- | --- | --- | --- | --- | --- | --- | --- |
| Parameters | EMINCA II  (V1, N=747) | Tamborini et al.  (V2, N=245) | D’Andrea et al.  (V3, N=250) | Addetia et al.  (V4, N=245) | Lakatos et al.  (V5, N=300) | P1  (V1 vs V2) | P2  (V1 vs V3) | P3  (V1 vs V4) | P4  (V1 vs V5) |
| 3D-RVEDV (ml) | 82.7±21.3 | 86±21 | 126.3±23.1 | 124±33 | NR | 0.035 | <0.001 | <0.001 | NR |
| 3D-RVEDVi (ml/m^2^) | 49.3±11.0 | 49±10 | 73.5±13.1 | 69±16 | 53.8±12.3 | 0.691 | <0.001 | <0.001 | <0.001 |
| 3D-RVESV (ml) | 37.3±11.3 | 29±11 | 56±18.3 | 55±17 | NR | <0.001 | <0.001 | <0.001 | NR |
| 3D-RVESVi (ml/m^2^) | 22.2±5.9 | 16±6 | 33.4±10.3 | 30±8 | 22.0±6.1 | <0.001 | <0.001 | <0.001 | 0.623 |
| 3D-RVEF (%) | 55.2±5.0 | 67±8 | 58.7±9.5 | 56±5 | 59.2±5.7 | <0.001 | <0.001 | 0.030 | <0.001 |

Data are analyzed by the Tomtec software in the EMINCA II and presented as mean ± SD.

| **Supplementary Table 7** Comparison of 3D-RV measurements in EMINCA II and by Maffessanti et al. | | | | | | |
| --- | --- | --- | --- | --- | --- | --- |
|  | EMINCAII | | | Maffessanti et al. | | |
| Parameter | ALL | Men | Women | ALL | Men | Women |
| 3D-RVEDV (ml) | 79 (55, 121) | 88 (61, 130) | 73 (53, 112) | 91 (61, 150) | 107 (74, 163) | 81 (58, 120) |
| 3D-RVESV (ml) | 36 (22, 58) | 40 (25, 64) | 33 (20, 51) | 35 (16, 72) | 44 (22, 80) | 30 (15, 52) |
| 3D-RVEF (%) | 55 (48, 64) | 54 (47, 62) | 55 (48, 64) | 62 (47, 77) | 60 (45, 75) | 63 (49, 79) |

Data are analyzed by the Tomtec software in the EMINCA II and presented as median (5th and 95th percentiles).

| **Supplementary Table 8** Comparison between 3D-RV and 3D-LV measurements in EMINCA II | | | |
| --- | --- | --- | --- |
|  | Right ventricle | Left ventricle | P value |
| EDV (mL) | 83.9±21.7 | 73.3±18.4 | <0.001 |
| EDVi (mL/m^2^) | 49.9±11.1 | 43.6±9.7 | <0.001 |
| ESV (mL) | 39.2±12.2 | 26.6±9.1 | <0.001 |
| ESVi (mL/m^2^) | 23.3±6.4 | 15.8±5.0 | <0.001 |
| SV (mL) | 46.0±11.3 | 46.7±11.9 | 0.127 |
| SVi (mL/m^2^) | 27.4±6.0 | 27.8±6.6 | 0.127 |
| EF (%) | 52.2±7.3 | 63.9±7.2 | <0.001 |

Data are analyzed by the Tomtec software and expressed as mean ± SD.

| **Supplementary Table 9** Inclusion and exclusion criteria of EMINCA II |
| --- |
| Inclusion criteria |
| Han nationality |
| ≥18 years of age |
| No history of cardiac disease, lung disease and kidney disease |
| No history of hypertension*, dyslipidemia* and diabetes |
| Normal blood pressure (systolic blood pressure [SBP] <140mmHg and diastolic blood pressure [DBP] <90mmHg) |
| Normal body mass index (18-28kg/m^2^) |
| Exclusion criteria |
| Pregnant or lactating women |
| Professional athletes |
| History of alcoholism |
| Any other major diseases (cardiovascular diseases, endocrine diseases, respiratory diseases, severe anemia, connective tissue disease, renal dysfunction, liver dysfunction, hyperlipidemia and more than mild valvular disease) |

*In the age groups of >65 years, a history of hypertension or hyperlipidemia was allowed as long as blood pressure and hyperlipidemia were well controlled with medications and there was no evidence of LV hypertrophy on echocardiography. This was caused by the difficulty in recruiting elderly volunteers with no history of hypertension and hyperlipidemia. At the end of enrollment, only 18 subjects were recruited under these conditions.
